# A case of epidemic myalgia with symptoms resembling acute purulent spondylitis and discitis

**DOI:** 10.1186/s12891-016-1181-x

**Published:** 2016-08-04

**Authors:** Tsuneaki Kenzaka, Yukariko Hida, Masanori Matsumoto, Hozuka Akita

**Affiliations:** 1 Division of Community Medicine and Career Development, Kobe University Graduate School of Medicine, 2-1-5, Arata-cho, Hyogo-ku, Kobe, Hyogo 652-0032 Japan; 2 Department of Internal Medicine, Hyogo Prefectural Kaibara Hospital, Tamba, Japan

**Keywords:** Coxsackievirus, Epidemic myalgia, Low back pain, Purulent spondylitis, Purulent discitis

## Abstract

**Background:**

Epidemic myalgia is a disease that presents with fever and extreme myalgia of the trunk due to an acute enterovirus infection. The trunk pain is mainly in the chest or in the epigastrium. We aimed to highlight a case of epidemic myalgia where initial diagnosis needed differentiation from acute purulent spondylitis and discitis.

**Case presentation:**

A 33-year-old woman presented with fever, chills, and acute episodes of low back pain. The sole unusual finding was pain upon spinal percussion, limited to the 4th and 5th lumbar vertebrae. Spinal MRI showed no abnormality. Paired serum samples from disease days 4 and 15 showed a significant increase in coxsackievirus B3–neutralizing antibodies. Based on this course, we diagnosed epidemic myalgia.

**Conclusions:**

Epidemic myalgia should be considered when differentiating acute low back pain accompanied by fever.

## Background

Epidemic myalgia is a disease that presents with fever and extreme myalgia of the trunk due to an acute enterovirus infection (mainly of coxsackievirus group B) [1]. The trunk pain is mainly in the chest or in the epigastrium. We aimed to highlight a case of epidemic myalgia due to coxsackievirus group B infection, for which the diagnosis needed to be differentiated from acute purulent spondylitis and discitis.

## Case presentation

**Fig. 1:**
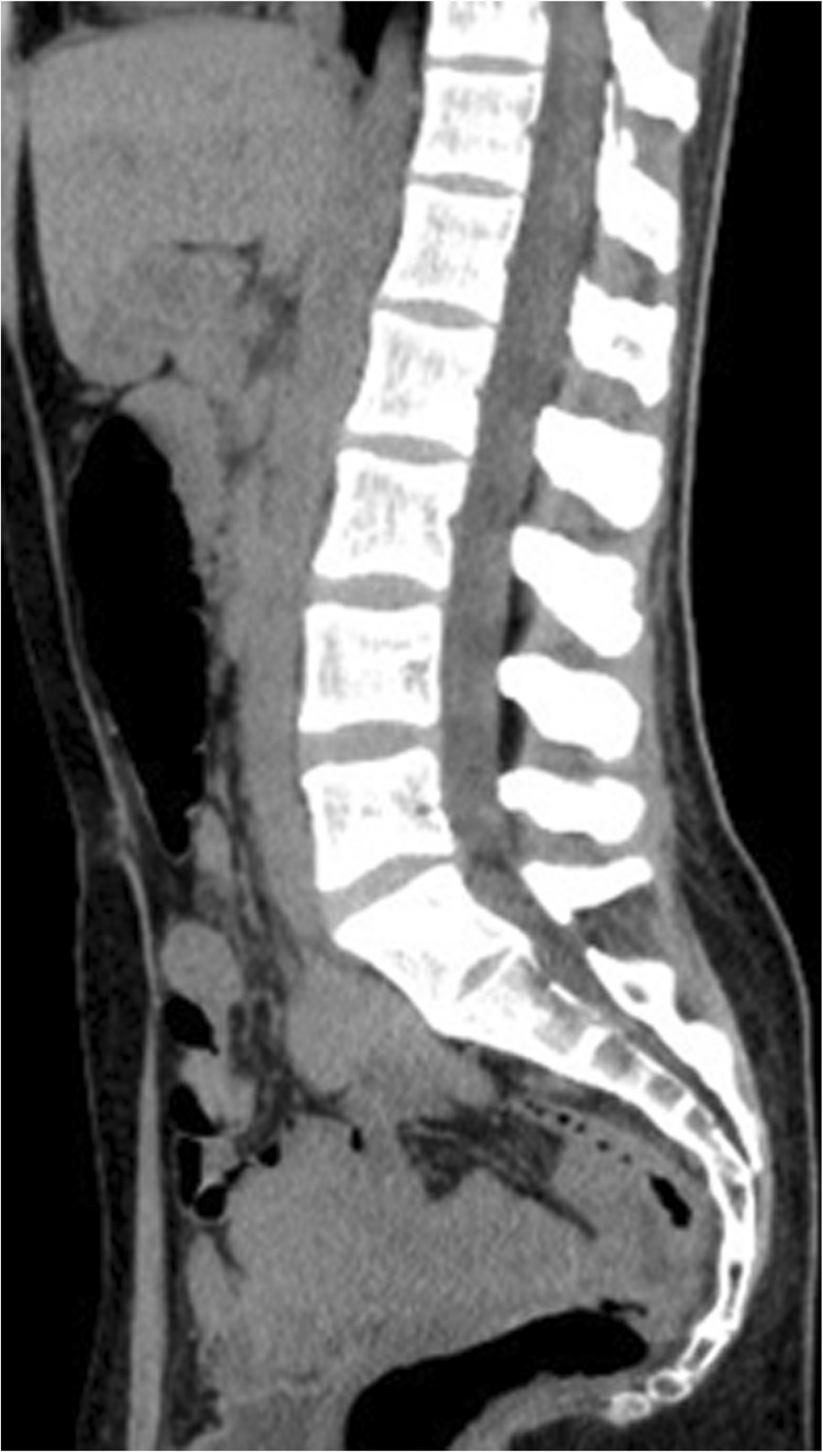
Abdominal and pelvic sagittal computed tomography (CT) image on disease day 2. CT showed no abnormalities

**Fig. 2:**
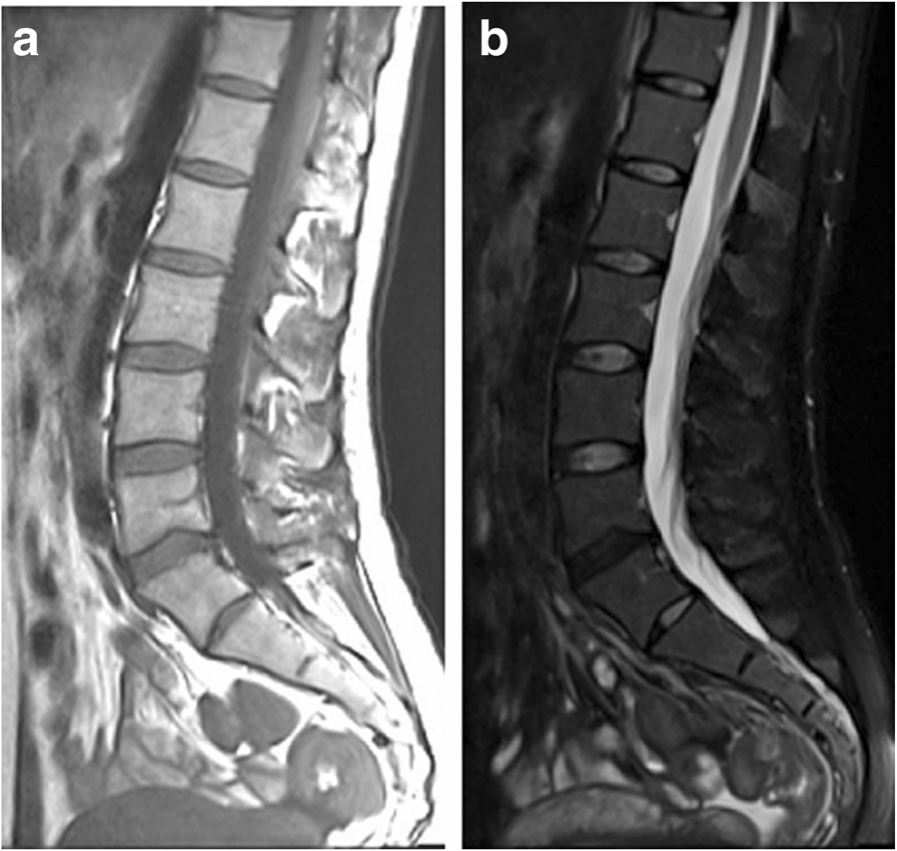
Spinal magnetic resonance imaging on disease day 7. **a** T1 weighted image and **b** short tau inversion recovery image. MRI showed no signs of acute purulent spondylitis, discitis, or acute inflammation

The patient was a 33-year-old woman with nothing of note in her medical history. Two weeks before admission, the patient’s 5-year-old son had a fever of approximately 38 °C and pharyngeal pain. The child was diagnosed with an acute upper respiratory tract infection, and his symptoms improved within a few days. Several days before our patient’s admission, she began experiencing pharyngeal pain. On the day she went for emergency outpatient care, the patient had begun experiencing fever with chills and acute episodes of low back pain. During examination, her consciousness was clear, blood pressure was 115/85 mmHg, heart rate was regular at 110 beats/minute, body temperature was 39.5 °C, and respiratory rate was 20 breaths/minute. Her head, neck, and throat were normal. Cardiac and respiratory sounds were normal. Abdominal examination was normal. Spinal pain upon percussion was limited to the 4th and 5th lumbar vertebrae. Neurological examination was normal. Laboratory findings from the first visit were as follows: leukocyte, 2350/μL; neutrophil, 1557/μL; lymphocyte, 416/μL; C-reactive protein (CRP), 0.76 mg/dL; procalcitonin, ≤0.05 ng/mL; aspartate aminotransferase, 21 U/L; alanine aminotransferase, 17 U/L; lactate dehydrogenase, 109 U/L; creatine phosphokinase (CK), 115 U/L. All other laboratory values were within normal range. Urinalysis results were normal. The acute episodes of lumbar pain were accompanied by high fever. Therefore, acute purulent spondylitis and discitis were suspected. Hospitalization was recommended, but the patient refused. Therefore, she was placed under closely monitored outpatient care. Loxoprofen was administered internally as an antipyretic analgesic. Two blood culture sets were negative, as was a urine culture. Another blood test showed that leukocyte levels were low and CRP levels had peaked from the first examination. Abdominal and pelvic CT performed on disease day 2 showed no abnormalities (Fig. 1). Spinal MRI, which greatly contributes to prompt diagnosis of acute purulent spondylitis and discitis in comparison to CT [2], was performed on disease day 7, and results suggested no signs of acute purulent spondylitis or discitis (Fig. 2). Her fever persisted at 38 °C or higher, but resolved on disease day 6. The low back pain would become particularly worse with movement. The pain made it difficult for the patient to get up, and hindered her daily life. However, the pain had mostly disappeared by disease day 8. Paired serum samples from disease days 4 and 15 exhibited a significant increase in coxsackievirus B3–neutralizing antibodies (Table 1). Based on this disease course, we diagnosed the patient as having epidemic myalgia. The patient made an uneventful recovery without any residual symptoms 6 months after this acute episode.

**Table 1:** Viral antibody titers

Virus	Type	Method	Day 4	Day 15
Coxsackievirus	A3	NT	4	8
	A4	NT	<4	<4
	A5	NT	16	32
	A6	NT	<4	4
	A7	NT	4	4
	A9	NT	<4	<4
	A10	NT	32	32
	B1	NT	64	64
	B2	NT	<4	<4
	B3	NT	4	128
	B4	NT	32	64
	B5	NT	32	64
	B6	NT	64	128
Echovirus	1	NT	<4	<4
	4	NT	<4	<4
	6	NT	16	32
	11	NT	16	16
	14	NT	<4	<4

*NT* neutralization

## Discussion

We presented a case of epidemic myalgia with acute low back pain, in which the initial diagnosis needed to be differentiated from acute purulent spondylitis and discitis. During the course of the disease, the leukocyte count decreased, the CRP test was negative, and no abnormal CT or MRI findings were observed. We diagnosed the patient as having epidemic myalgia based on the upper respiratory tract infection that occurred in the patient’s family, on the presence of fever and pain localized to the lower lumbar vertebrae, and on a significant increase in coxsackievirus group B–neutralizing antibodies in a paired serum sample. We could find no other case reports on epidemic myalgia with low back pain.

The pain seen in epidemic myalgia is thought to be caused by local viral proliferation in the muscles of the chest, diaphragm, abdomen, and other areas [1]. The area of pain is often larger than the palm of a hand and can occur unilaterally or bilaterally around the costal bone margins. Intermittent intensification of the pain is typical of the disease, and the pain can be exacerbated by body movements and breathing. Frequent concomitant symptoms that have been reported include fever (97 %), pharyngitis (85 %), headache (50 %), gastrointestinal disorders (50 %), chest wall pressure pain (25 %), otitis (25 %), dermatitis (25 %), and testicular pain (10 %) [3]. Nasal discharge and cough usually do not occur. The chest pain needs to be differentiated from acute coronary syndrome, as sudden episodes of left chest pain accompanied by electrocardiographic changes can occur [4, 5]. Differentiation from acute abdomen is also necessary in cases with epigastric pain, and, especially, hypochondrium pain [1]. Moreover, periumbilical, and hypogastric, pain is sometimes present [1]. This case of epidemic myalgia had low back pain mimicking acute purulent spondylitis and discitis. Epidemic myalgia should be considered when differentiating acute low back pain accompanied by fever.

This disease was first described by Ejnar Sylvest in the 1930s, when he reported cases from the Danish island of Bornholm [6]. It is possible that the reason why acute low back pain has not been previously reported as a symptom of epidemic myalgia is that the disease was first described many years ago, which may have limited the number of further reports regarding novel symptoms. Moreover, there is low awareness regarding this disease in East Asia [7, 8]. Furthermore, this disease is difficult to recognize, and thus many cases go unreported [7]. Finally, non-steroidal anti-inflammatory drugs are effective against epidemic myalgia pain [3]. In the case presented here, loxoprofen helped relieve the pain, and the symptoms disappeared by disease day 8.

Laboratory findings typically show almost normal leukocyte count [9]. CRP and CK are normal or slightly elevated [9]. In addition, ultrasound, radiography, and abdominal computed tomographic scans are normal in patients with epidemic myalgia [9]. There are no systematic concepts of epidemic myalgia in MRI [10]. Several patients showed acute inflammation in MRI [10]. However, patients with epidemic myalgia have normal or slightly elevated CRP and CK, and the pain typically lasts 1 to 4 days [9], although pain lasting as long as 45 days has been described [7]. Therefore, MRI may show normal findings in many of patients with epidemic myalgia. In fact, MRI of this case performed on disease day 7 (during which her symptoms were almost gone) showed no signs of acute inflammation.

Coxsackievirus infections can be diagnosed with an antibody test, but such tests are generally not performed because the disease improves relatively quickly.

Thus, it is likely that epidemic myalgia is frequently misdiagnosed.

## Conclusion

We presented a case of epidemic myalgia with acute low back pain, in which the initial diagnosis needed to be differentiated from acute purulent spondylitis and discitis. Epidemic myalgia should be considered when differentiating acute low back pain accompanied by fever.

## Abbreviations

CK, creatine phosphokinase; CRP, C-reactive protein; CT, computed tomography; MRI, magnetic resonance imaging

## References

[1] Bennett JE, Dolin R, Blaser MJ (2014). Mandell, Douglas, and Bennett’s principles and practice of infectious diseases, ed 8.

[2] Leone A, Dell’Atti C, Magarelli N, et al. Imaging of spondylodiscitis. Eur Rev Med Pharmacol Sci. 2012;16(Suppl 2):8–19. 22655479

[3] Schmidt NJ, Magoffin RL, Lennette EH (1973). Association of group B Coxsackie viruses with cases of pericarditis, myocarditis, or pleurodynia by demonstration of immunoglobulin M antibody. Infect Immun.

[4] McConaghy JR, Oza RS (2013). Outpatient diagnosis of acute chest pain in adults. Am Fam Physician.

[5] Hussein L, Al-Rawi H: Chest wall myositis in a patient with acute coronary syndrome. BMJ Case Rep. doi:10.1136/bcr-2014-206664. 10.1136/bcr-2014-206664 PMC4195170 25312897

[6] Williams WO (1961). Bornholm disease survey 1956, 1957 and 1958. J Coll Gen Pract.

[7] Huang WT, Lee PI, Chang LY (2010). Epidemic pleurodynia caused by coxsackievirus B3 at a medical center in northern Taiwan. J Microbiol Immunol Infect.

[8] Nishino Y (2001). Epidemic pleurodynia. Ryoikibetsu Shokogun Shirizu.

[9] Ivan Y, Kelly BP (2014). Bornholm disease: case report and a review of the literature. Infect Dis Clin Pract (Baltim Md).

[10] Mizuta K, Kuroda M, Kurimura M (2012). Epidemic myalgia in adults associated with human parechovirus type 3 infection, Yamagata, Japan, 2008. Emerg Infect Dis.

